# Assessment of Potentially Toxic Elements Pollution and Human Health Risks in Polluted Farmland Soils around Distinct Mining Areas in China—A Case Study of Chengchao and Tonglushan

**DOI:** 10.3390/toxics11070574

**Published:** 2023-06-30

**Authors:** Qi Leng, Dajun Ren, Zhaobo Wang, Shuqin Zhang, Xiaoqing Zhang, Wangsheng Chen

**Affiliations:** 1 College of Resource and Environmental Engineering, Wuhan University of Science and Technology, Wuhan 430081, China; wust201701144071@163.com (Q.L.); 13377854423@163.com (Z.W.); zhangshuqin@wust.edu.cn (S.Z.); zhangxiaoqing@wust.edu.cn (X.Z.); chenwangsheng@wust.edu.cn (W.C.); 2 Hubei Key Laboratory for Efficient Utilization and Agglomeration of metallurgic Mineral Resources, Wuhan University of Science and Technology, Wuhan 430081, China

**Keywords:** index of geoaccumulation, pollution investigation, pollution index, potential ecological risk, hazard index

## Abstract

This research study investigates the extent of heavy metal pollution and pollution trends in agricultural soil in mining areas during different time periods. A total of 125 soil samples were collected from two mining areas in China, the Chengchao iron mine and Tonglushan ancient copper mine. The samples were analyzed for various potentially toxic elements (PTEs). The index of geoaccumulation (Igeo), pollution index ($P_{i}$), potential ecological risk index ($E_{r}^{i}$), and hazard index (HI) were calculated to evaluate the pollution status of PTEs in the farmland around the two mining areas. The sources of PTEs were inferred by pollution distribution, and the pollution conditions of the two mining areas were compared. The results showed that the pollution of ancient copper mines was relatively severe. The main pollution elements were Cu, Cd, and As, and their average $P_{i}$ values were 3.76, 4.12, and 1.84, respectively. These PTEs mainly came from mining and transportation. There are no particularly polluted elements in the Chengchao iron mine and the average $P_{i}$ of all PTEs were classified as light pollution and had a wide range of sources. The findings suggest that the ancient copper mine, due to outdated mining techniques and insufficient mine restoration efforts, resulted in the spread and accumulation of PTEs in the soil over an extended period, making the farmland soil around the ancient copper mine more polluted compared to the Chengchao iron mine. In the two mining areas, there is no risk of cancer for adults and children. However, the RI values of Cr in adults and children are higher than 10^−4^, which indicates that the carcinogenic risk of Cr in these soils is very high. The non-carcinogenic effects of PTEs on the human body in the soil of ancient copper mine are also higher than that of the Chengchao iron mine.

## 1. Introduction

Developing countries face severe soil PTEs pollution [1]. PTEs are significant harmful trace elements [2,3]. Behaviors such as open-pit mining will produce tailings ponds and abandoned mines, bringing PTEs pollution to the soil [4]. Some studies have also shown that Cu and Pb in tailings are easily transferred in the soil, causing many farmland soils around them to be polluted through infiltration and migration in the soil [5,6,7]. These PTEs penetrate farmland soil and bring not only PTEs to crops but also affect the water environment and even pollute the water source for daily human drinking. Environmental problems have become more severe in recent years, most notably the degradation of ecosystems due to the accumulation of PTEs [8,9,10,11]. Excessive industrial activities such as non-ferrous metal mining and smelting, agricultural activities such as chemical fertilizers and herbicides, and PTEs migration driven by vehicle traffic have introduced large amounts of PTEs into the environment [12]. Nigeria has reported numerous casualties caused by PTEs pollution from illegal and unprofessional mining practices [13]. Cd, Zn, and Pb pollution were found in the soil of Ganhe Industrial Park in the upper reaches of the Yellow River in China [14]. Heavy metal pollution has also been found in the soil of agricultural fields in China’s coastal triangle, all of which come from long-term petroleum extraction and smelting emissions [15]. Heavy metal pollution has also been found in the rice soil and rice soil and locally produced grains (rice) around the abandoned high-arsenic coal mine area in Xingren County, southwest China [16].

The polluted farmland soil will affect the production of crops and thus affect human health [17]. Crops, animals, and humans readily absorb PTEs through the biological chain, among which cadmium rice is the most known [18]. PTEs in the soil will accumulate in the human body through the food chain, resulting in various diseases [19]. For example, long-term accumulation of Cd can lead to lung adenocarcinoma, renal failure, and fracture. Long-term accumulation of Hg can affect the human nerve center. And typical accumulation of Cu has been shown to lead to kidney injury, liver injury, Wilson’s disease and dermatitis, or chronic asthma [8,20,21]. The accumulation of PTEs can also lead to changes in the soil’s physical and chemical properties, thereby affecting the activities of soil microbial communities and destroying the soil’s self-healing ability [22]. A human health risk assessment of soil samples collected in the Hongshaquan coal mine waste dump and artificial forest in Xinjiang found that the main exposure pathways were ingestion and dermal contact, and the contribution of Pb and As to noncarcinogenic risk was higher [23]. In evaluating the risks and sources of potentially toxic elements in typical rare-earth mining areas in southern China, Fan Jiajia et al. [24] found that the Pb from traffic pollution and rare-earth mining contributed the most to the non-carcinogenic risk at the site, accounting for 26.81% and 40.35% of the risk for adults and children.

PTEs pollution is a significant problem worldwide [25]. For example, the concentration of Mn in wastewater-irrigated farmland in Albaminci, Ethiopia, reached 190.65 mg·Kg^−1^ [26]. Fe, Mn, Ni, and Cu concentrations of 36,000 mg·Kg^−1^, 1285 mg·Kg^−1^, 1025 mg·Kg^−1^, and 54.3 mg·Kg^−1^ were detected in soils of Zhob and Loralai valleys in Baluchistan, Pakistan [27]. The concentrations of Zn, Pd, and Cd detected in farmland soil in northern India were 44.43 ± 11.29 mg·Kg^−1^, 14.62 ± 4.31 mg·Kg^−1^, and 2.71 ± 2.32 mg·Kg^−1^, respectively [28]. PTEs pollution was found in the soil around a coal mining area in the city of Tai’an, China [29].

The objectives of this research are as follows: (1) the concentration and distribution of PTEs (Cd, As, Pb, Cr, Cu, Ni, Zn) in the farmland soil around the mining area were determined. (2) Use various pollution indices (Igeo, $P_{i}$, $P_{N}$, ${E_{r}}^{i}$, RI) and human health risks of PTEs to evaluate the pollution of agricultural soil in the two mining areas [30,31,32]. (3) Use ArcGIS geographic information system software to analyze the overall distribution trend of PTEs in the soil around the mining area to estimate the source of pollution.

## 2. Materials and Methods

### 2.1. Sample Collection Plan and Area

This study was conducted at the Tonglushan ancient copper mine in Huangshi City and the Chengchao iron mine in Ezhou City, Hubei Province in China. With both mines as the center, there are many villages nearby. Almost every village has a large amount of farmland and various agricultural products. The mining and smelting period of the ancient copper mine site in Tonglushan began in the Shang Dynasty (16th to 11th centuries BC). It continued through the Western Zhou Dynasty, the Spring and Autumn Warring States Period, to the Han Dynasty, lasting over a thousand years. The industrial and mining activities in the bronze mining area of Tonglushan have already affected the area’s ecological environment. The accumulated PTEs in the soil pose a potential threat to residents. Chengchao iron mine is located at 114°32′~115°05′ east longitude and 30°00′~30°06′ north latitude. The Chengchao iron mine project was completed and began operating in November 1969. The design capacity is 1.5 million tons of iron ore annually and has been expanded to 3 million tons. The production scale ranks the top three among the similar mines in China, and it is a critical ore production base in WISCO. In the past 50 years, many PTEs may have been discharged into the surrounding farmland while mining and transporting minerals. Therefore, this article will compare the pollution levels of these two mining areas of different eras and explore the sources of pollution.

**Figure 1:**
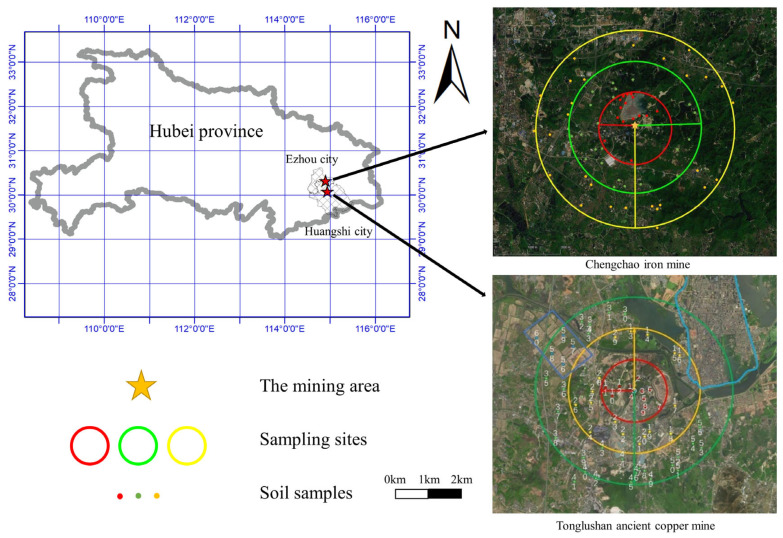
Schematic diagram of sampling points in the study area.

We collected 125 soil samples in the farmland around the mining area, as shown in Figure 1, and divided the research area into an inner circle (0~1 km), middle circle (1~2 km), and outer circle (2~3 km). Samples were taken on farmland or farms in villages in the research area, and soil samples were randomly collected at 0~20 cm in the farmland tillage layer by a serpentine distribution method. We marked the area where the soil was located and GPS information, took pictures, and recorded the collection location. Finally, 5 kg of soil sampled at each point by the four-point method was returned to the laboratory for further treatment.

### 2.2. Chemical Analysis

We spread the collected soil away from direct sunlight for natural air-drying, picked out the stones and plants in the soil, ground the soil, passed it through a 100-mesh sieve, and put the treated soil powder into plastic bags. The bags were marked with the determination of soil PTEs concentration.

This study determined the total concentrations of Cd, As, Pb, Cr, Cu, Ni, and Zn in soil samples. These seven PTEs belonged to the priority PTEs pollutants designated by the US EPA [33]. The soil was digested by microwave using the aqua regia microwave digestion method [34]. After the digested soil solution was filtered by a syringe equipped with a water filter head, the concentrations of seven PTEs in the surface soil were determined by inductively coupled plasma mass spectrometry (ICP-MS,7900, Thermo Electron Corporation). The method detection limits of metals were in the range of 0.02–0.16 mg kg^−1^.

Quality control and quality assurance (QC/QA) procedures were performed. For each group of 5 samples, a blank and a matrix sample are used to calculate the accuracy. Each soil sample was analyzed in triplicate. The relative standard deviations (RSDs) were all between 0.06% and 15.2%. The standard deviation of matrix samples (S.D.) was less than 5%.

### 2.3. Pollution Evaluation of Heavy Metal

The concentration of PTEs such as Cd, As, Pb, Cr, Cu, Ni, and Zn in soil samples was evaluated by the geoaccumulation index (Igeo) method to evaluate the PTEs pollution status of agricultural land in this area.

The calculation formula of Igeo is:

(1) $$Igeo=log_{2}\;\left(\frac{C_{n}}{1.5B_{n}}\right)$$

where $C_{n}$ is the measured concentration of element *n*, and $B_{n}$ is the background content of element *n* in Chinese soils [35]. A constant of 1.5 accounted for potential changes in baseline data [36]. As shown in Table S1, the geoaccumulation index consists of seven classes or grades.

The concentration of PTEs such as Cd, As, Pb, Cr, Cu, Ni, and Zn in soil samples were jointly evaluated by the single-factor pollution index method ($P_{i}$) and the Nemerow comprehensive pollution index method ($P_{N}$). PTEs pollution status.

The formula for calculating $P_{i}$ is:

(2) $$P_{i}=\frac{C_{i}}{S_{i}}$$

The formula for calculating $P_{N}$ is:

(3) $$P_{N}=\sqrt{\frac{{P_{\mathrm{avg}}}^{2}+{P_{imax}}^{2}}{2}}$$

where $C_{i}$ is the measured value of pollutant mg kg^−1^; the risk screening value of $S_{i}$ for pollutants mg kg^−1^. The risk screening value and background value of PTEs at each pH are shown in Table S2. $P_{\mathrm{avg}}$ is the average of all single-factor pollution indices; $P_{imax}$ is the maximum of each single-factor pollution index. The index evaluation criteria are shown in Table S3.

### 2.4. Potential Ecological Risk Index

The concentration of PTEs such as Cd, As, Pb, Cr, Cu, Ni, and Zn in soil samples was evaluated by the Hakanson potential ecological risk index method.

The formula for calculating $E_{r}^{i}$ is:

(4) $${E_{r}}^{i}=P_{i}\times {T_{r}}^{i}$$

The formula for calculating the RI is:

(5) $$RI=\sum {E_{r}}^{i}$$

where $P_{i}$ is the single factor pollution index of PTEs element i, and ${T_{r}}^{i}$ i is the corresponding toxicity coefficient of PTEs element i, as shown in Table S4.

### 2.5. Human Health

The health risk assessment of PTEs in soil is widely used to quantify the carcinogenic and non-carcinogenic risks to humans through ingestion, inhalation, skin, and dietary exposure. The average daily dose (mg Kg^−1^ day^−1^) of potentially toxic metals (mg Kg^−1^ day^−1^) for adults and children through ingestion (*ADD_ing_*), skin contact (*ADD_derm_*), and inhalation (*ADD_inh_*) was calculated through Equations. The calculation formula is as follows:

Ingestion:

(6) $$ADD_{ing}=\frac{C_{soil}\times IngR\times EF\times ED}{BW\times AT}\times 10^{-6}$$

Inhalation:

(7) $$ADD_{inh}=\frac{C_{soil}\times InhR\times EF\times ED}{PEF\times BW\times AT}$$

Dermal adsorption:

(8) $$ADD_{derm}=\frac{C_{soil}\times AF\times SA\times ABS\times EF\times ED}{BW\times AT}\times 10^{-6}$$

The non-carcinogenic hazards of a single PTEs are usually characterized by the hazard quotient (*HQ*):

(9) $$HQ=\frac{ADD}{RfD}$$

The total exposure hazard index (*HI*) method is used. *HI* is the sum of each *HQ*:

(10) $$HI=\sum HQ=\sum \frac{ADD}{RfD}$$

The carcinogenic hazards of a single PTEs are expressed in *CR*:

(11) CR=ADI×SF

The total carcinogenic hazard is expressed in *CR_T_*:

(12) $$CR_{T}=\sum CR$$

Tables S7 and S8 show the exposure factors and values used to estimate intake and risk.

### 2.6. Data Analysis

The spatial distribution of PTEs was analyzed using a geographic information system. The average value, range value, and standard deviation of PTEs in farmland soil around the two mining areas were sorted out. In addition, to confirm the comparison with the standard, the soil pollution was analyzed by Igeo, $P_{i}$ and $E_{r}^{i}$ values. Moreover, calculate the non-carcinogenic and carcinogenic risk values of the human health risk index, and evaluate the harmfulness of PTEs contaminated soil to humans.

## 3. Results and Discussion

### 3.1. Concentration of PTEs

The statistics of PTEs content around the two mining areas are shown in Table 1. The concentrations of seven PTEs (Cd, As, Pb, Cr, Cu, Ni, and Zn) were determined around the two mining areas.

These values were compared with the Chinese soil background concentrations and quality guidelines. In addition to the Cr concentration, the concentration of other PTEs in the ancient copper mines is higher than that of the Chengchao iron mines. The average concentrations of PTEs in ancient copper mines were higher than the background values of soil in China, especially Cd and Cu, which were 21 times and 16 times the background values, respectively. They had far exceeded farmland soil’s Cd and Cu concentrations around a polymetallic mine in South China [37]. However, in the average concentration of PTEs in the Chengchao iron mine, Pb and Ni did not exceed the background value, among which only the concentration of Cu and Zn exceeded twice the background value. These results preliminarily show that the degree of pollution in the ancient copper mine is more severe than that of the Chengchao iron mine, which was in the mining stage.

Chengchao iron mine is in the “young adult” period of mining, and soil pollution has not yet appeared. At the same time, after long-term mining, the ancient copper mine has been abandoned, and its pollution of the surrounding soil has gotten out of hand. On the other hand, compared with modern mining technology, ancient mining technology lacks more scientific pollution control technology. In particular, the failure to pay attention to the restoration work after mining has caused the PTEs pollution index of the ancient copper mine to be much higher than that of the Chengchao iron mine.

### 3.2. Evaluation of Soil Pollution

#### 3.2.1. Geoaccumulation Index Evaluation

**Figure 2:**
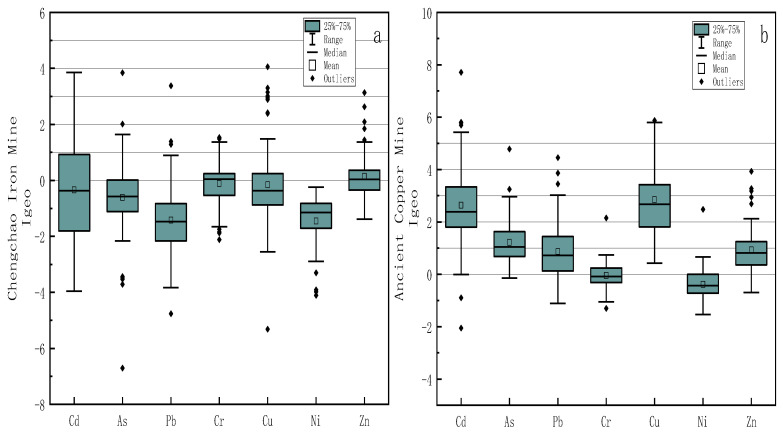
Box plots of the geoaccumulation index (Igeo) values for PTEs in Chengchao Iron Mine (**a**) and Ancient Copper Mine (**b**).

The Igeo values of PTEs in farmland soil around these two mining areas are shown in Figure 2. An Igeo value above 1.0 indicates increased PTEs content and soil pollution [38]. The mean Igeo of the Chengchao iron mine increases in the order of Ni < Pb < As < Cd < Cu < Cr < Zn; the mean Igeo of the ancient copper mine increases in the order of Ni < Cr < Pb < Zn < As < Cd < Cu. The trends of the two mining areas were quite different. Nevertheless, the ancient copper mine is similar to the trend found in Liberty State Park in the United States [39]. According to the pollution level corresponding to Igeo in Table S1 and combined with the judgment of [1] on Igeo, only the mean Igeo of Zn in the Chengchao iron mine was unpolluted to moderately polluted. It is much higher than the average Igeo (−6.93) of Zn in surface sediments along the coast of the Bizerte Sea in Tunisia [40], but it is similar to the Igeo (Zn) in the soil along the Gulf of Bengal [41]. In contrast, the Igeo of other PTEs in the Chengchao iron mine was practically unpolluted. Nevertheless, in the ancient copper mine, the mean Igeo of Cu and Cd were moderate to strongly polluted, the mean Igeo of As was moderate pollution, and the mean Igeo of Zn and Pb were unpolluted to moderately polluted. In addition, the mean Igeo of Ni and Cr were practically unpolluted. The Igeo (Cd) in the soil of the ancient copper mine site is similar to the Igeo (Cd) in the soil and stream sediments of an abandoned artisanal small-scale gold mine in Betare-Oya, Cameroon [42], and both belong to the highest mean Igeo in the measured PTEs. These data show that the pollution of the Chengchao iron mine to the surrounding farmland was generally low. In contrast, PTEs pollution of the ancient copper mine was more severe than that of the Chengchao iron mine, and more types of PTEs caused pollution.

#### 3.2.2. Evaluation of Single Factor Pollution Index and Comprehensive Pollution Index

**Figure 3:**
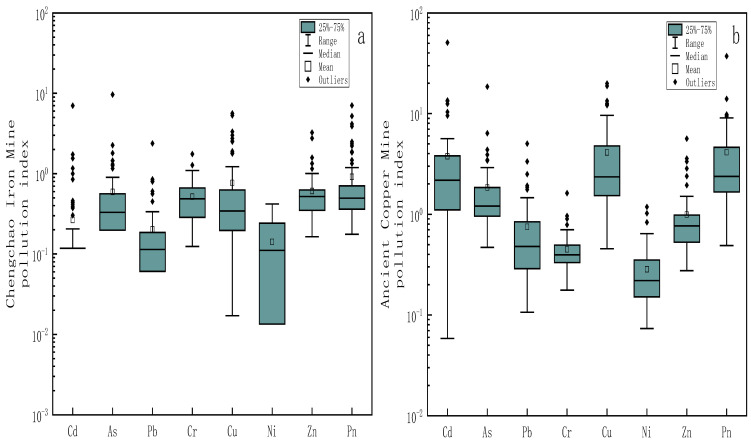
Box plots of log-transformed pollution index ($P_{i}$) and comprehensive pollution index ($P_{N}$) values for PTEs in Chengchao Iron Mine (**a**) and Ancient Copper Mine (**b**).

As shown in the box diagram in Figure 3, the mean values of $P_{i}$ of various PTEs in the Chengchao iron mine increased in the order of Ni < Pb < Cd < Cr < As < Zn < Cu, while the mean values of $P_{i}$ of various PTEs in the ancient copper mine increased in the order of Ni < Cr < Pb < Zn < As < Cd < Cu. Compared with the arrangement law of the average Igeo of PTEs, the Chengchao iron mine was quite different, and the mean value of $P_{i}$ of Cu was the highest, while the order of the ancient copper mine did not change. The mean values of $P_{i}$ of various PTEs in the Chengchao iron mine belonged to no pollution ($P_{i}$ < 1). However, of the mean $P_{i}$ values of various PTEs in the ancient copper mine, only the mean $P_{i}$ values of Ni, Cr, Pb, and Zn belonged to the non-polluting state ($P_{i}$ < 1), which were 0.28, 0.45, 0.75, and 0.99, respectively. The mean $P_{i}$ of As was 1.84, belonging to light pollution (1 ≤ $P_{i}$ < 2); the mean $P_{i}$ of Cd and Cu were 3.76 and 4.12, which belonged to heavy pollution (3 ≤ $P_{i}$). Judging the comprehensive pollution of various PTEs by the $P_{N}$ values, the $P_{N}$ values of the Chengchao iron mine ranged from 0.18 to 7.04, with a mean value of 0.91, which indicated that most of the soil of the Chengchao iron mine was uncontaminated. Nevertheless, there was a small amount of soil with a $P_{N}$ value as high as 7.04, which was severe pollution. The $P_{N}$ values of the ancient copper mine ranged from 0.49 to 37.30, and the mean value reached 4.14, which indicated that the soil pollution of the ancient copper mine was quite severe, the mean value had reached a state of heavy pollution, and the maximum value of $P_{N}$ was as high as 37.30. Compared with the Chengchao iron mine, the pollution of the ancient copper mine was particularly serious. The $P_{N}$ value obtained by [13] in the soil of Sao Paulo (Paris) was 25.4. The $P_{N}$ value obtained by [43] in the soil of the city park of Guangzhou Province (China) was 7.9. The $P_{N}$ value of the ancient copper mine, as high as 37.30, is more polluted than the soil standard of the city park.

**Figure 4:**
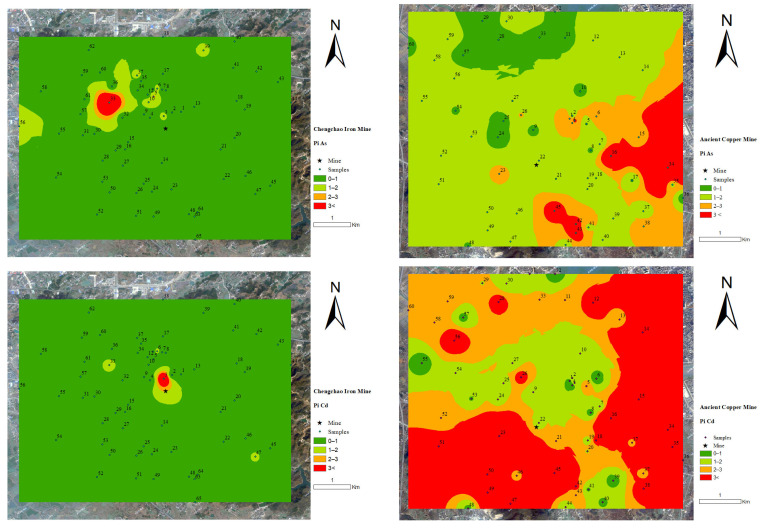

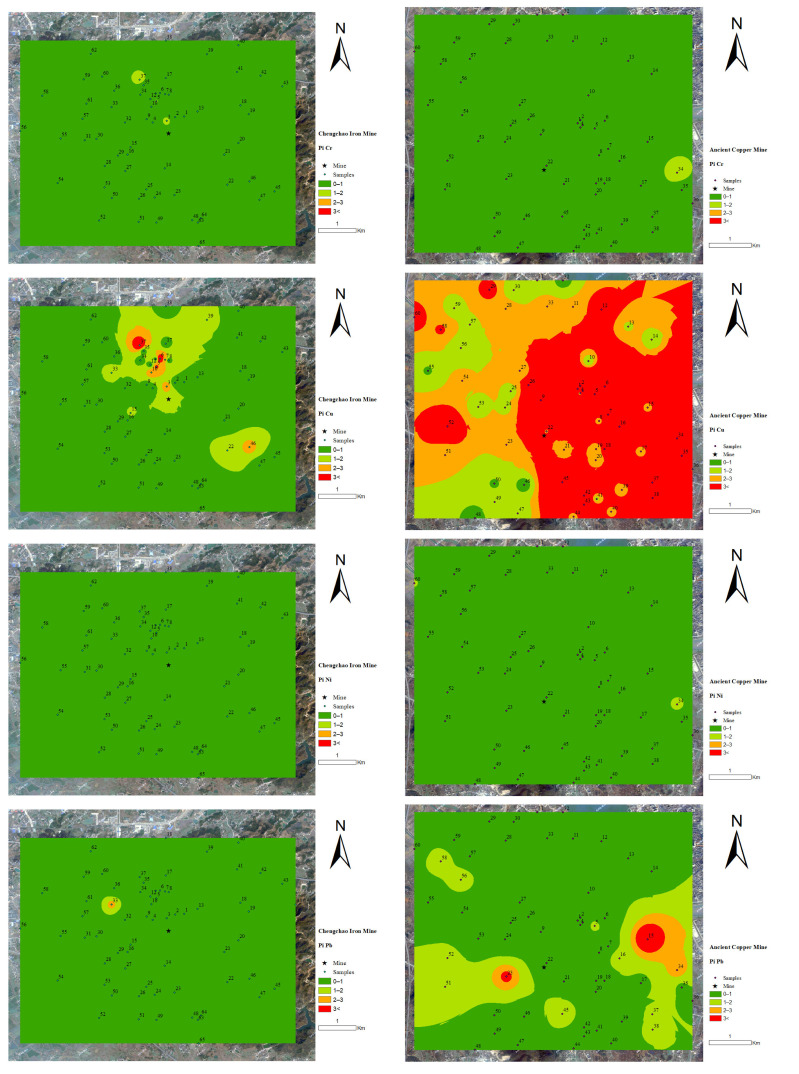

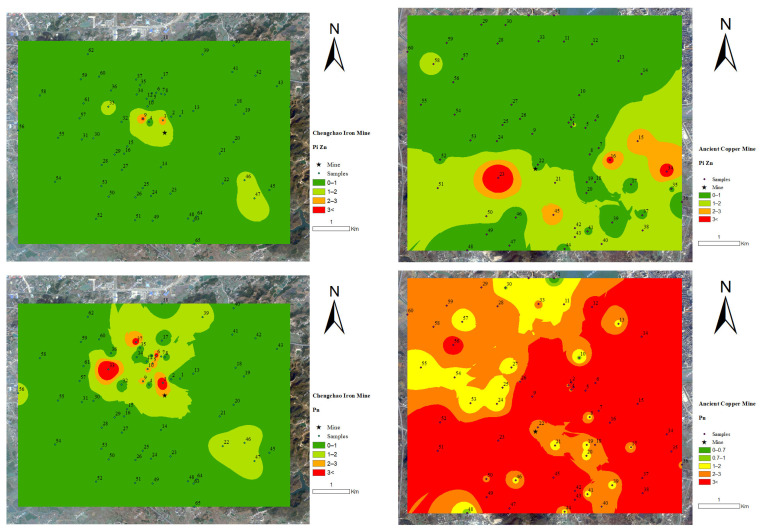
Spatial distribution maps of pollution index ($P_{i}$) and comprehensive pollution index ($P_{N}$) values.

In Figure 4, the inverse distance weight method in ArcMap 10.2 was used to analyze the spatial interpolation of the PTEs pollution index and Nemerow comprehensive pollution index of the Chengchao iron mine and ancient copper mine. From the spatial distribution map of the Chengchao iron mine, $P_{Pb}$, $P_{As}$,$\;P_{Zn}$,$\;P_{Cr}$, and $P_{Cu}$ were similar to $P_{N}$, as shown in Table 2 and Table 3. First of all, we tested the normality of the experimental data and passed the Kolmogorov–Smirnov test (KS test). The results show that the data of $P_{i}$ and $P_{N}$ does not satisfy the normal distribution. Therefore, we used the Spearman correlation coefficient to perform a correlation analysis of the $P_{i}$ and $P_{N}$ of Chengchao iron mine and ancient copper mine. At the 0.01 level, there was a very significant positive correlation between $P_{Pb}$, $P_{As}$,$\;P_{Zn}$,$\;P_{Cr}$, and $P_{Cu}$ in the soil near the Chengchao iron mine and ancient copper mine. The pollution of the Chengchao iron mine was mainly distributed in the northwest of the mining area. Through on-site observation, it was found that the more polluted areas were all in the villages west of the tailing reservoir in the north of the mine site, which could be inferred to be caused by human activities transferring the soil around the tailing reservoir to farmland. The pollution of As and Pb in the far northwest of the mining area was more serious, different from the pollution in the mining area. This pollution may come from the household garbage of the villages at the sampling point. Compared with the distribution map of the Chengchao iron mine, the PTEs pollution of the ancient copper mine was severe. The pollution was mainly distributed in the eastern and southern areas of the mining area. The eastern part of the ancient copper mine was mostly cities and traffic roads, and the southern part was mainly from factories. These pollutions were all caused by human activities. The pollution in the northwestern suburbs was relatively light but still higher than the overall pollution of the Chengchao iron mine. It can be seen from Figure 4 that Cr and Ni have no or minimal pollution impact on the two mining areas.

#### 3.2.3. Potential Ecological Index Risk Assessment

**Figure 5:**
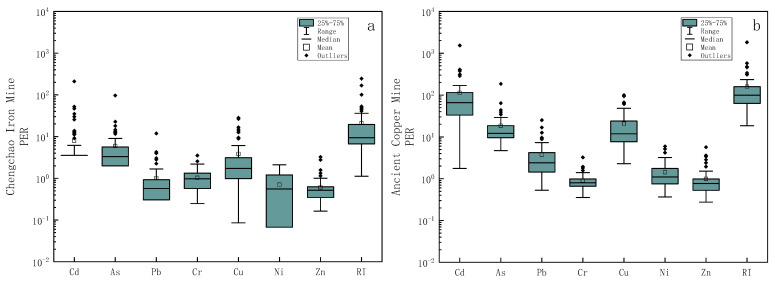
Box plots of log-transformed potential ecological risk index ($E_{r}^{i}$ and RI) values for PTEs in Chengchao Iron Mine (**a**) and Ancient Copper Mine (**b**).

The PER value reflects the potential ecological risks of hazardous elements to biological communities [1]. The single-factor potential ecological risk index and the comprehensive potential ecological risk index were used to evaluate the potential ecological risks of PTEs in the soil around the two mining areas as the ${E_{r}}^{i}$ values and RI values. As shown in the box plots in Figure 5, the mean values of various PTEs in the Chengchao iron mine ${E_{r}}^{i}$ were in ascending order as Zn < Ni < Pb < Cr < Cu < As < Cd, while the mean values of various PTEs in ancient copper mine ${E_{r}}^{i}$ in ascending order were Cr < Zn < Ni < Pb < As < Cu < Cd. The mean values of ${E_{r}}^{i}$ of all PTEs elements in the Chengchao iron mine were less than 40, and the potential ecological risk was evaluated as low risk. Among all the PTEs in ancient copper mines, only Cd has a mine values of ${E_{r}}^{i}$ of 112.84 (80 ≤ ${E_{r}}^{i}$ < 160), which could be evaluated as medium risk. In addition, the RI values of the Chengchao iron mine ranged from 1 to 246, with a mean value of 21, which belonged to low risk. But the RI value of the ancient copper mine ranged from 18 to 1836, with a mean value of 159, which belonged to medium risk. The RI value obtained in the soil of the city park of Bydgoszcz (Poland) [44] was 313. The RI value obtained in the soil of Faisalabad City (Pakistan) [45] was 93. The maximum RI value of the ancient copper mine is as high as 1836, which is more polluted than the soil standards of these two city parks. The mean value (159) is also higher than the urban park soil of Faisalabad City (Pakistan).

**Figure 6:**
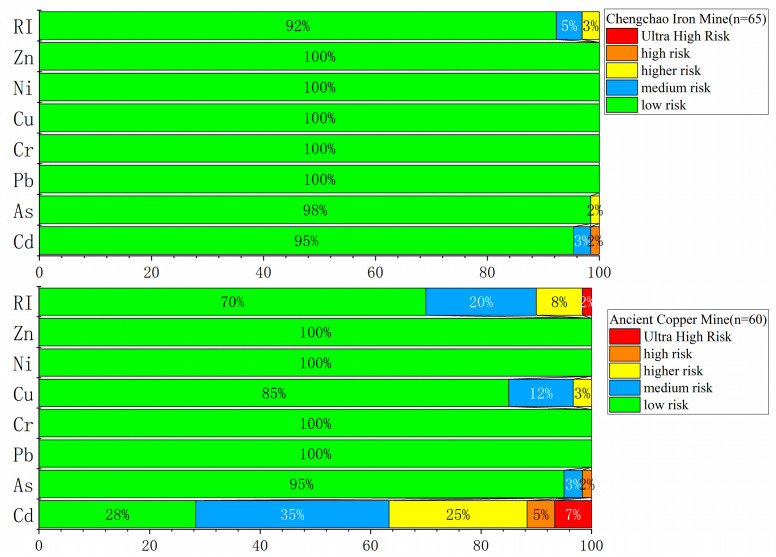
Stacked distribution chart of log-transformed potential ecological risk index ($E_{r}^{i}$ and RI) values for PTEs.

Figure 6 shows the proportion of potential ecological risk indices of different PTEs (As, Cd, and Cu, for example) in the total sample. Cd was the metal with the more variable potential ecological risk index values for both mines and, therefore, we decided to focus our comments on it. In the Chengchao iron mine, 95% of the sample soils were at low potential ecological risks, 3% were at medium risk, and 2% were at high risk. In the ancient copper mine, 28% of the sample soils were at low potential ecological risks, 35% of the sample soils were at medium potential ecological risks, and 25% of the sample soils were at high potential ecological risks. Further, 5% of the sample soils were at high potential ecological risk and 7% were at ultra-high potential ecological risk. Overall, Cd was important in affecting potential ecological risks in the two mining areas [46]. The impact of Cd on crops must be addressed. Most of the farmland around the mining area was dominated by rice cultivation, and the most common pollution present was “cadmium rice” [47]. Rice is the staple food in most countries. Therefore, cadmium pollution significantly impacts humans [48].

### 3.3. Human Health Assessment

There are numerous ways that the PTEs can enter the human body upon occupational or environmental exposure; specifically, they can be ingested, inhaled, and absorbed through the skin, as shown in Tables S9 and S10, similar to the results found in the soil of a typical metallurgical mining area in southern China by Zhou Lingfeng et al. [49]. Our research has found that the importance of three pathways to PTEs is significantly reduced (intake > skin contact > inhalation), and intake is the primary exposure route for most PTEs. Also, intake for children is higher than for adults, as the main soil ingestion occurs by hand-to-mouth behavior. In Chengchao’s study, the average daily As intake of children reached 0.36 mg kg^−1^ d^−1^, while the average daily As intake of adults through intake and absorption was 2.93 × 10^−2^ mg kg^−1^ d^−1^. In contrast, the average daily intake of As by children through inhalation and skin absorption is estimated to be 1.92 × 10^−5^ mg kg^−1^ d^−1^ and 2.73 × 10^−3^ mg kg^−1^ d^−1^, respectively. In the study of the ancient copper mine site, the average daily As intake of children through intake reached 1.04 mg kg^−1^ d^−1^, while the average daily As intake of adults through the intake pathway was 8.47 × 10^−2^ mg kg^−1^ d^−1^. Our research also found that in Chengchao iron ore, the non-carcinogenic risk values of As for adults and children were 3.66 × 10^−2^ and 3.63 × 10^−1^, respectively, and the non-carcinogenic risk values of Cr for adults and children were 0.1 and 0.234, respectively. In addition, the non-carcinogenic risk values of other PTEs are less than 1. In the ancient copper mine site, the non-carcinogenic risk values of As for adults and children were 0.106 and 1.05, respectively, and the non-carcinogenic risk values of Cr for adults and children were 9.87 × 10^−2^ and 0.23, respectively. Unlike Chengchao iron ore, the non-carcinogenic risk value of As for children in the ancient copper mine site was 1.05. In addition, the non-carcinogenic risk value of other PTEs is less than 1. According to the classification groups defined by the International Agency for Research on Cancer (IARC), Pb, Zn, and Cu can be regarded as non-carcinogenic elements [50]; therefore, only Cr, Cd, and Ni have carcinogenic risks. The carcinogenic risk of Chengchao iron mine soil research area to adults is 1.02 × 10^−7^ (Cd), 1.58 × 10^−5^ (As), 4.05 × 10^−7^ (Cr) and 7.05 × 10^−10^ (Ni); for children, it is 3.14 × 10^−7^ (Cd), 1.50 × 10^−5^ (As), 5.58 × 10^−7^ (Cr) and 3.16 × 10^−10^ (Ni). The carcinogenic risk of the soil research area of the ancient copper mine site to adults is 2.06 × 10^−6^ (Cd), 4.55 × 10^−5^ (As), 3.97 × 10^−7^ (Cr), and 1.90 × 10^−9^ (Ni); for children, it is 5.83 × 10^−6^ (Cd), 3.99 × 10^−5^ (As), 5.06 × 10^−7^ (Cr), and 7.85 × 10^−10^ (Ni). In the two mining areas, there was no RI of PTE for adults and children higher than 10^−4^, which indicates that there was no carcinogenic risk. The non-carcinogenic and carcinogenic risk value of PTEs in the soil of the ancient copper mine site to the human body is higher than that of the Chengchao iron mine.

## 4. Conclusions

This study compared the overall composition and pollution levels of PTEs in the soil around two mining areas in China of different ages. In the ancient copper mine, the Igeo,$\;P_{i}$ and $E_{r}^{i}$ values of Cu, Cd, and As are the highest, and their sources mainly come from the water transfer of the soil in the mining area and the dust settlement brought about by transportation. However, in the Chengchao iron mine, the PTEs with the highest values of Igeo,$\;P_{i}$ and $E_{r}^{i}$ are not fixed, and only Cu is always in a high state. It can be seen that mining in the mining area pollutes the farmland around the Chengchao iron mine, but the impact is relatively lower than that of the ancient copper mine. Most importantly, the mean $P_{N}$ of the ancient copper mine (4.14), which is much larger than the mean $P_{N}$ of Chengchao iron mine (0.91). Furthermore, from the analysis of the Gis map, it can be seen that the PTEs pollution in the farmland soil around the ancient copper mining area is more expansive, and the concentration is higher. In addition, according to the human health risk assessment results, the soil around the ancient copper mine is obviously severely polluted by PTEs. This soil pollution poses many carcinogenic and non-carcinogenic risks to the public. From the above results, due to the backwardness of ancient mining technology and the lack of a standardized environmental protection system for mining areas, the degree of pollution of farmland soil in ancient copper mining areas is much higher than that of modern mining areas, such as the Chengchao iron mine. At the same time, soil transfer in mining areas is also the leading cause of the spread of PTEs pollutants in surrounding farmland. Therefore, by comparing the pollution levels of different mining areas, this study shows the importance of improving the protection system of mining areas. At the same time, more reference data have been added to the soil assessment situation in Hubei Province, China.

The limitation of this study is that there are more soils around mining areas in Hubei, China that have not been collected and evaluated. When more and larger areas of mining area soil are tested and evaluated, we will get more complete soil PTEs monitoring data. These data can provide a reference for our future studies on pollution control.

## Figures and Tables

**Table 1:** Concentration summary of individual PTEs (mg kg^−1^) around mining area (*n* = 125).

Metals	Chengchao Iron Mine (*n* = 65)				Ancient Copper Mine (*n* = 60)			
	Median	Maximum	Minimum	Mean ± SD	Median	Maximum	Minimum	Mean ± SD
Cd	<LOQ	2.10	<LOQ	0.10 ± 0.3	0.76	30.5	0.04	2.00 ± 4.32
As	10.5	241	<LOQ	17.4 ± 31.4	34.6	464	15.1	49.7 ± 62.3
Pb	13.3	405	<LOQ	24.9 ± 51.9	64.6	854	18.1	109 ± 144
Cr	94.5	264	21.1	97.6 ± 52.6	86.3	405	37.1	96.6 ± 48.8
Cu	26.2	565	0.85	62.5 ± 101	213	1990	45.5	361 ± 430
Ni	14.4	34.2	<LOQ	13.2 ± 9.87	29.9	225	13.9	36.1 ± 28.5
Zn	115	976	42.6	152 ± 145	196	1700	68.7	277 ± 283

Soil environmental quality risk control standard for soil contamination of agricultural land (GB 15618-2018).

**Table 2:** Spearman correlation coefficients among $P_{i}$ and $P_{N}$ of PTEs in soils around Chengchao iron mine.

$P_{i}$	Cd	As	Pb	Cr	Cu	Ni	Zn	$P_{N}$
Cd	1.000							
As	0.199	1.000						
Pb	0.288 *	0.318 **	1.000					
Cr	−0.006	0.456 **	0.477 **	1.000				
Cu	0.196	0.511 **	0.689 **	0.784 **	1.000			
Ni	−0.059	0.087	0.028	0.581 **	0.267 *	1.000		
Zn	0.171	0.306 *	0.626 **	0.777 **	0.797 **	0.353 **	1.000	
$P_{N}$	0.218	0.565 **	0.570 **	0.791 **	0.840 **	0.313 *	0.863 **	1.000

** Correlation is significant at the 0.01 level (2-tailed). * Correlation is significant at the 0.05 level (2-tailed).

**Table 3:** Spearman correlation coefficients among $P_{i}$ and $P_{N}$ of PTEs in soils around ancient copper mine.

$P_{i}$	Cd	As	Pb	Cr	Cu	Ni	Zn	$P_{N}$
Cd	1.0000							
As	0.340 **	1.0000						
Pb	0.633 **	0.403 **	1.0000					
Cr	0.388 **	0.226	0.578 **	1.0000				
Cu	0.261 *	0.340 **	0.356 **	0.172	1.0000			
Ni	0.286 *	−0.051	0.493 **	0.843 **	0.140	1.0000		
Zn	0.542 **	0.547 **	0.796 **	0.511 **	0.503 **	0.309 *	1.0000	
$P_{N}$	0.664 *	0.512 **	0.517 **	0.365 **	0.718 **	0.126	0.626 **	1.0000

** Correlation is significant at the 0.01 level (2-tailed). * Correlation is significant at the 0.05 level (2-tailed).

## Data Availability

The datasets used and analyzed during the current study are available from the corresponding author upon reasonable request.

## Supplementary Materials

The following supporting information can be downloaded at: https://www.mdpi.com/article/10.3390/toxics11070574/s1, Table S1: Pollution grade of heavy metal accumulation index; Table S2: Soil pollution risk screening value of agricultural land; Table S3: Graded evaluation of heavy metal pollution index; Table S4: Toxicity Correspondence Coefficient of Required Heavy Metal; Table S5: Hierarchical evaluation of potential ecological risks of heavy metals; Table S6: Related guideline values of heavy metal concentration (mg/kg); Table S7: Definition and reference of some parameters for health risk assessment of heavy metal in soils; Table S8: Reference dose (RfD, mg/(kg d)) and slope factor (SF, (kg d)/mg) of toxic elements for health risk assessment.; Table S9: The hazard index of PTEs in the soil around Chengchao Iron Mine; Table S10: The hazard index of PTEs in the soil around ancient copper mine; [51,52,53,54,55,56,57] are cited in the Supplementary Materials.
