# Supplementary material for: Assessment of Potentially Toxic Elements Pollution and Human Health Risks in Polluted Farmland Soils around Distinct Mining Areas in China—A Case Study of Chengchao and Tonglushan
Source: Toxics. 2023 Jun 30;11(7):574. doi: 10.3390/toxics11070574 (PMC10385012; doi:10.3390/toxics11070574)
Supplement: Supplementary file 1 [file toxics-11-00574-s001.zip › toxics-2455741-supplementary.pdf]

Supplementary Material

Table of Contents

Table S1 Pollution grade of heavy metal accumulation index

Table S2 Soil pollution risk screening value of agricultural land

Table S3 Graded evaluation of heavy metal pollution index

Table S4 Toxicity Correspondence Coefficient of Required Heavy Metal

Table S5 Hierarchical evaluation of potential ecological risks of heavy metals

Table S6 Related guideline values of heavy metal concentration (mg/kg)

Table S7 Definition and reference of some parameters for health risk assessment of heavy metal in soils

Table S8 Reference dose (RfD, mg/(kg•d)) and slope factor (SF, (kg•d)/mg) of toxic elements for health risk assessment.

Table S9. The hazard index of PTEs in the soil around Chengchao Iron Mine.

Table S10. The hazard index of PTEs in the soil around ancient copper mine.

Table S1 Pollution grade of heavy metal accumulation index

Grade	*Igeo*[a]	Class of pollution
I	<0	practically unpolluted
II	0-1	unpolluted to moderately polluted
III	1-2	moderately polluted
IV	2-3	moderately to strongly polluted
V	3-4	strongly polluted
VI	4-5	strongly to extremely polluted
VII	5<	extremely polluted

[a.] Qing X, Yutong Z, Shenggao L. 2015. Assessment of heavy metal pollution and human health risk in urban soils of steel industrial city (Anshan), Liaoning, Northeast China. Ecotoxicology and Environmental Safety. 120:377–385. doi:10.1016/j.ecoenv.2015.06.019.

Table S2 Soil pollution risk screening value of agricultural land

	Metals	Background[a] (mg kg^{-1})	Risk Screening Values[b] (mg kg^{-1})			
			pH≤5.5	5.5<pH≤6.5	6.5<pH≤7.5	pH>7.5
1	Cd	0.097	0.3	0.3	0.3	0.6
2	As	11.2	40	40	30	25
3	Pb	26.0	70	90	120	170
4	Cr	61.0	150	150	200	250
5	Cu	22.6	50	50	100	100
6	Ni	26.9	60	70	100	190
7	Zn	74.2	200	200	250	300

[a.] CNEMC.1990.

[b.] Soil environmental quality risk control standard for soil contamination of agricultural land (GB 15618-2018)

Table S3 Graded evaluation of heavy metal pollution index[a]

Grade	P_i	pollution assessment	P_N	pollution assessment
I	$P_i<1$	no pollution	$P_N \leq 0.7$	Safety
II	$1\leq P_i<2$	light pollution	$0.7< P_N \leq 1.0$	Vigilant
III	$2\leq P_i<3$	Moderately polluted	$1.0< P_N \leq 2.0$	light pollution
IV	$P_i \geq 3$	heavy pollution	$2.0< P_N \leq 3.0$	Moderately polluted
V			$P_N>3.0$	heavy pollution

The single-factor pollution index method evaluates that as long as one pollutant exceeds the standard, the soil sample is considered to exceed the standard, and the main heavy metals and their harm levels can be determined; while the Nemerow comprehensive pollution index method considers a single factor with the most serious pollution. In the weighting process, the influence of subjective factors in the weight coefficient is avoided, and the soil environmental quality of the agricultural land can be reflected more comprehensively.

[a.] Wu W, Wu P, Yang F, Sun D, Zhang D-X, Zhou Y-K. 2018. Assessment of heavy metal pollution and human health risks in urban soils around an electronics manufacturing facility. Science of The Total Environment. 630:53–61. doi:10.1016/j.scitotenv.2018.02.183

Table S4 Toxicity Correspondence Coefficient of Required Heavy Metal[a]

Project	Cd	As	Pb	Cr	Cu	Ni	Zn
$T_r{}^i$	30	10	5	2	5	5	1

[a.] HAKANSON L.An ecological risk index for aquatic pollution control.a sedimentological approach[J].Water research,1980,14(8):975-1001

Table S5 Hierarchical evaluation of potential ecological risks of heavy metals[a]

Grade	E_r^i	RI	potential ecological risk
I	E_r^i<40	RI<150	low risk
II	40≤E_r^i<80	150≤RI<300	medium risk
III	80≤E_r^i<160	300≤RI<600	higher risk
IV	160≤E_r^i<320	600≤RI<1200	high risk
V	E_r^i≥320	RI≥1200	ultra high risk

The single-factor potential ecological risk index can reflect the risk degree of each heavy metal, and the comprehensive potential ecological risk index can reflect the comprehensive risk of multiple heavy metals.

[a.] HAKANSON L.An ecological risk index for aquatic pollution control.a sedimentological approach[J].Water research,1980,14(8):975-1001

Table S6 Related guideline values of heavy metal concentration (mg/kg)

Symbol	Cr	Cu	Zn	As	Cd	Ni	Pb
Background of China[a]	61.0	22.6	74.2	11.2	0.097	26.9	26.0
Chinese soil quality gudielines[b]	200	100	250	30.0	0.30	50	300
Toxic response factors[c]	2	5	1	10	30	6	5

a CNEMC (China National Environmental Monitoring Center) *The Background Concentrations of Soil Elements of China*; China Environmental Science Press: Beijing, 1990;

b MEP and Ministry of Land and Resources (MLR) *MEP and MLR announce the report on national general survey on soil contamination;* MEP and Ministry of Land and Resources (MLR):2014;

Table S7 Definition and reference of some parameters for health risk assessment of heavy metal in soils

Calculation formula	Definition	Unit	Value		Reference
			Children	Adults	
ABS	Dermal absorption factor	unitless	0.001（non-carcinogenic）<br>0.01（carcinogenic）		USDOE(2011)
AT	Average time	days	365 x ED (non-carcinogenic)<br>365× 70（carcinogenic）		MEP (2014)
AF	Skin adherence factor	mg/cm day	0.2	0.7	MEP (2014)
BW	Body weight	kg	15.9	56.9	USEPA (2001)
EF	Exposure frequency	days/year	350	350	USEPA (2001)
ED	Exposure duration	years	6	24	USDOE(2011)
IngR	Ingestion rate of soil	mg/day	103	30	USDOE(2011)
InhR	Inhalation rate of soil	m^3/day	7.5	15	USDOE(2011)
PEF	Inhalation factor for emission particles	m^3/kg	1.36×10^9	1.36×10^9	Environmental site assessment guideline (2009)
SA	Surface area of the skin	cm^2	1600	4350	Environmental site assessment guideline (2009)

Table S8 Reference dose (RfD, mg/(kg•d)) and slope factor (SF, (kg•d)/mg) of toxic elements for health risk assessment.

Parameters	As	Cd	Cr	Cu	Hg	Ni	Pb	Zn
R_fD_{ing}	3.00×10^{-4} [a]	1.00×10^{-3} [a]	3.00×10^{-3} [a]	0.04 [a]	3.00×10^{-4} [a]	0.02 [a]	3.50×10^{-3} [a]	0.30 [b,c]
R_fD_{inh}	3.01×10^{-4} [a]	1.00×10^{-3} [a]	2.86×10^{-5} [a]	4.02×10^{-2} [a]	2.40×10^{-5} [a]	2.06×10^{-2} [a]	3.52×10^{-3} [a]	0.30 [b,c]
R_fD_{dermal}	1.23×10^{-4} [a]	1.00×10^{-5} [a]	6.00×10^{-5} [a]	1.20×10^{-2} [a]	2.10×10^{-5} [a]	5.40×10^{-3} [a]	5.25×10^{-4} [a]	6.00×10^{-2} [b,c]
SF_{ing}	1.50 [d]	6.10 [e]	8.50×10^{-3} [e]	–	–		8.50×10^{-3} [e]	–
SF_{inh}	15.1 [d]	6.30 [e]	42 [e]	–	–	0.84 [e]		–
SF_{dermal}	3.66 [d]		–	–	–		–	–

a Men, C., Liu, R.M., Wang, Q.R., Miao, Y.X., Wang, Y.F., Jiao, L.J., Li, L., Cao, L.P., Shen, Z.Y., Li, Y., Crawford, D., 2021. Spatial-temporal characteristics, source-specific variation and uncertainty analysis of health risks associated with heavy metals in road dust in Beijing, China. Environmental Pollution 278, 116866;

b Wang, X.Y., Liu, E.F., Lin, Q., Liu, L., Yuan, H.Z., Li, Z.J., 2020. Occurrence, sources and health risks of toxic elements in road dust from a mega city (Nanjing) in China. Environmental Pollution 263, 114518;

c Penteado, J.O., Brum, R.D., Ramires, P.F., Garcia, E.M., dos Santos, M., da Silva, F.M.R., 2021. Health risk assessment in urban parks soils contaminated by metals, Rio Grande city (Brazil) case study. Ecotoxicology and Environmental Safety 208, 111737;

d Ma, L., Xiao, T.F., Ning, Z.P., Liu, Y.Z., Chen, H.Y., Peng, J.Q., 2020. Pollution and health risk assessment of toxic elements in soils under different land use in sulphide mineralized areas. Science of the Total Environment 724, 138176;

e Huang, J.L., Wu, Y.Y., Sun, J.X., Li, X., Geng, X.L., Zhao, M.L., Sun, T., Fan, Z.Q., 2021. Health risk assessment of heavy elements in park soils of the largest megacity in China by using Monte Carlo simulation coupled with Positive matrix factorization model. Journal of Hazardous Materials 415, 125629.

Table S9. The hazard index of PTEs in the soil around Chengchao Iron Mine.

PTEs		Cd	As	Pb	Cr	Cu	Ni	Zn
HQ_{ing} (mean)	adult	4.90×10^{-5}	2.93×10^{-2}	3.59×10^{-3}	1.64×10^{-2}	7.90×10^{-4}	3.33×10^{-4}	2.55×10^{-4}
	children	6.01×10^{-4}	0.36	4.41×10^{-2}	2.02×10^{-1}	9.70×10^{-3}	4.09×10^{-3}	3.14×10^{-3}
HQ_{inh} (mean)	adult	1.80×10^{-8}	1.07×10^{-5}	1.31×10^{-6}	6.34×10^{-4}	2.89×10^{-7}	1.19×10^{-7}	9.39×10^{-8}
	children	3.22×10^{-8}	1.92×10^{-5}	2.35×10^{-6}	1.13×10^{-3}	5.17×10^{-7}	2.13×10^{-7}	1.68×10^{-7}
HQ_{derm} (mean)	adult	8.28×10^{-5}	7.26×10^{-3}	2.43×10^{-3}	8.34×10^{-2}	2.67×10^{-4}	1.25×10^{-4}	1.30×10^{-4}
	children	1.87×10^{-4}	2.73×10^{-3}	9.14×10^{-4}	3.14×10^{-2}	1.00×10^{-4}	4.71×10^{-5}	4.88×10^{-5}
HI	adult	1.32×10^{-4}	3.66×10^{-2}	6.02×10^{-3}	0.1	1.06×10^{-3}	4.58×10^{-4}	3.85×10^{-4}
	children	7.88×10^{-4}	3.63×10^{-1}	4.50×10^{-2}	2.34×10^{-1}	9.80×10^{-3}	4.14×10^{-3}	3.19×10^{-3}
CR_T	adult	1.02×10^{-7}	1.58×10^{-5}		4.05×10^{-7}		7.05×10^{-10}	
	children	3.14×10^{-7}	1.50×10^{-5}		5.58×10^{-7}		3.16×10^{-10}	

Table S10. The hazard index of PTEs in the soil around ancient copper mine.

PTEs		Cd	As	Pb	Cr	Cu	Ni	Zn
HQ_{ing} (mean)	adult	9.82×10^{-4}	8.47×10^{-2}	1.56×10^{-2}	1.61×10^{-2}	4.84×10^{-3}	8.98×10^{-4}	4.62×10^{-4}
	children	1.21×10^{-2}	1.04	1.91×10^{-1}	1.98×10^{-1}	5.95×10^{-2}	1.10E-02	5.68×10^{-3}
HQ_{inh} (mean)	adult	3.61×10^{-7}	3.10×10^{-5}	5.69×10^{-6}	6.22×10^{-4}	1.77×10^{-6}	3.21×10^{-7}	1.70×10^{-7}
	children	6.46×10^{-7}	5.55×10^{-5}	1.02×10^{-5}	1.11×10^{-3}	3.17E $\times 10^{-6}$	5.74×10^{-7}	3.04×10^{-7}
HQ_{derm} (mean)	adult	1.66×10^{-3}	2.10×10^{-2}	1.05×10^{-2}	8.19×10^{-2}	1.64×10^{-3}	3.38×10^{-4}	2.35×10^{-4}
	children	3.75×10^{-3}	7.89×10^{-3}	3.96×10^{-3}	3.08×10^{-2}	6.16×10^{-4}	1.27×10^{-4}	8.83×10^{-5}
HI	adult	2.64×10^{-3}	1.06×10^{-1}	2.61×10^{-2}	9.87×10^{-2}	6.48×10^{-3}	1.24×10^{-3}	6.97×10^{-4}
	children	1.58×10^{-2}	1.05	1.95×10^{-1}	0.23	6.01×10^{-2}	1.12×10^{-2}	5.77×10^{-3}
CR_T	adult	2.06×10^{-6}	4.55×10^{-5}		3.97×10^{-7}		1.90×10^{-9}	
	children	5.83×10^{-6}	3.99×10^{-5}		5.06×10^{-7}		7.85×10^{-10}	
